# Performance assessment of a NaI(Tl) gamma counter for PET applications with methods for improved quantitative accuracy and greater standardization

**DOI:** 10.1186/s40658-015-0114-3

**Published:** 2015-05-06

**Authors:** Martin A Lodge, Daniel P Holt, Paul E Kinahan, Dean F Wong, Richard L Wahl

**Affiliations:** Division of Nuclear Medicine, The Russell H. Morgan Department of Radiology and Radiological Sciences, Johns Hopkins University School of Medicine, Baltimore, MD USA; Department of Radiology, University of Washington, Seattle, WA USA; Department of Radiology, Washington University School of Medicine, St. Louis, MO USA

**Keywords:** Gamma counter, Well counter, Positron emission tomography, Efficiency, Calibration, Sample volume, Standardization

## Abstract

**Background:**

Although NaI(Tl) gamma counters play an important role in many quantitative positron emission tomography (PET) protocols, their calibration for positron-emitting samples has not been standardized across imaging sites. In this study, we characterized the operational range of a gamma counter specifically for positron-emitting radionuclides, and we assessed the role of traceable ^68^Ge/^68^Ga sources for standardizing system calibration.

**Methods:**

A NaI(Tl) gamma counter was characterized with respect to count rate performance, adequacy of detector shielding, system stability, and sample volume effects using positron-emitting radionuclides (409- to 613-keV energy window). System efficiency was measured using ^18^F and compared with corresponding data obtained using a long-lived ^68^Ge/^68^Ga source that was implicitly traceable to a national standard.

**Results:**

One percent count loss was measured at 450 × 10^3^ counts per minute. Penetration of the detector shielding by 511-keV photons gave rise to a negligible background count rate. System stability tests showed a coefficient of variation of 0.13% over 100 days. For a sample volume of 4 mL, the efficiencies relative to those at 0.1 mL were 0.96, 0.94, 0.91, 0.78, and 0.72 for ^11^C, ^18^F, ^125^I, ^99m^Tc, and ^51^Cr, respectively. The efficiency of a traceable ^68^Ge/^68^Ga source was 30.1% ± 0.07% and was found to be in close agreement with the efficiency for ^18^F after consideration of the different positron fractions.

**Conclusions:**

Long-lived ^68^Ge/^68^Ga reference sources, implicitly traceable to a national metrology institute, can aid standardization of gamma counter calibration for ^18^F. A characteristic feature of positron emitters meant that accurate calibration could be maintained over a wide range of sample volumes by using a narrow energy window centered on the 511-keV peak.

## Background

Historically, NaI(Tl) gamma counters have been an important instrument in nuclear medicine [1], and they continue to play a key role in modern practice. Clinical applications include *in vitro* studies of glomerular filtration rate, cerebrospinal fluid leak, red cell mass, and plasma volume [2]. Gamma counters are also widely used in conjunction with positron emission tomography (PET), typically in the research setting. PET applications include metabolite analysis [3], tracer kinetic studies [4], and pre-clinical biodistribution studies [5]. While quantitative accuracy is important in any setting, PET applications typically have slightly different quantitative requirements than clinical *in vitro* studies. Many *in vitro* tests involve comparison of the count rate obtained from patient samples with similar measurements of a reference source. Relative quantification of this sort can lead to an elegant elimination of errors, but it is frequently not applicable to PET studies where absolute quantification is often required. For example, in biodistribution studies, the radioactivity concentration in blood or tissue samples needs to be measured in absolute units (e.g., Bq/mL). Tracer kinetic studies require only that the gamma counter be calibrated in the same units as the PET images, which could potentially be in arbitrary units. However, much complexity can be avoided, particularly at sites that have multiple PET systems, if all instruments are calibrated in absolute units. In order to obtain gamma counter measurements with the required accuracy, careful isotope-specific calibration and an awareness of the operational range of the instrument are essential.

The technical performance required for gamma counters intended for use with positron emitters differs slightly from the requirements for lower energy single-photon emitters. The NaI(Tl) crystal thickness needs to be optimized for 511-keV photon detection. The shielding surrounding the detector needs to be sufficient to eliminate the penetrative 511-keV emissions from nearby sources such as samples in adjacent rack positions. The count rate performance of the system needs to be adequate to accommodate the relatively high activities encountered in blood samples obtained during the early phase of kinetic studies. And while stable performance is desirable in all applications, it is particularly important for absolute quantification because sample counting may occur some time from the last system calibration. An additional consideration that affects studies involving both single-photon emitters and positron emitters is the loss of sensitivity with increasing sample volume [6]. When liquid samples are involved, this effect can usually be minimized by consistently counting samples of equal volume. However, this approach may not be feasible when counting solid tissue samples [7,8] such as those obtained during pre-clinical biodistribution studies. Sample volume effects are, therefore, another important performance parameter when gamma counters are to be used with samples of potentially different sizes.

Another key consideration for PET applications is calibration accuracy. Whereas radionuclide calibrators (commonly referred to as dose calibrators) produce measurements that are directly presented in units of activity (e.g., Bq), gamma counter results are typically given in arbitrary units (counts per minute (CPM)). In order to convert these data into units of activity, users require an estimate of the efficiency of the gamma counter for the isotope of interest. Manufacturers often provide tables of efficiency values for various common isotopes, but the accuracy of these data can be uncertain or the appropriate measurement conditions may not be clearly defined. Analytic models have been developed to calculate efficiency [9], but these models are probably better suited to examining design trade-offs, rather than for predicting the efficiency of a specific device with high accuracy. Experimental measurement with reference to a radionuclide calibrator is a practical way of estimating efficiency, although this approach requires a meticulous experimental technique. Experimental errors can be introduced at numerous stages in a procedure that involves radionuclide calibrator measurement, phantom preparation, pipetting, sample weighing, and counting. Furthermore, experimental measurements of gamma counter efficiency are limited by the accuracy of the radionuclide calibrator. Recent developments have seen the introduction of ^68^Ge/^68^Ga sources that are specifically intended to aid calibration of radionuclide calibrators used in PET applications [10]. The activity in these sources is known with high accuracy and is traceable to a National Institute of Standards and Technology (NIST) standard. Additionally, the geometry of the source has been designed to mimic a clinical syringe, and cross-calibration factors have been developed to account for the different decay characteristics of ^68^Ge/^68^Ga and ^18^F. The use of these traceable sources has led to increased standardization and improved quantitative accuracy of radionuclide calibrators for ^18^F. While these ^68^Ge/^68^Ga mock syringe sources are generally not compatible with gamma counters due to their size, companion sources designed for use with NaI(Tl) gamma counters have recently become available. These new ^68^Ge/^68^Ga gamma counter reference sources are implicitly traceable to an NIST standard and may help eliminate much of the experimental error associated with gamma counter calibration for positron-emitting isotopes.

In this study, we characterized the technical performance of a commercial well-type NaI(Tl) gamma counter, specifically for use with positron-emitting radiopharmaceuticals. As part of this evaluation, we assessed the extent to which traceable ^68^Ge/^68^Ga sources can be used to optimize instrument calibration, potentially leading to greater consistency of performance across sites.

## Methods

### Gamma counter

**Figure 1:**
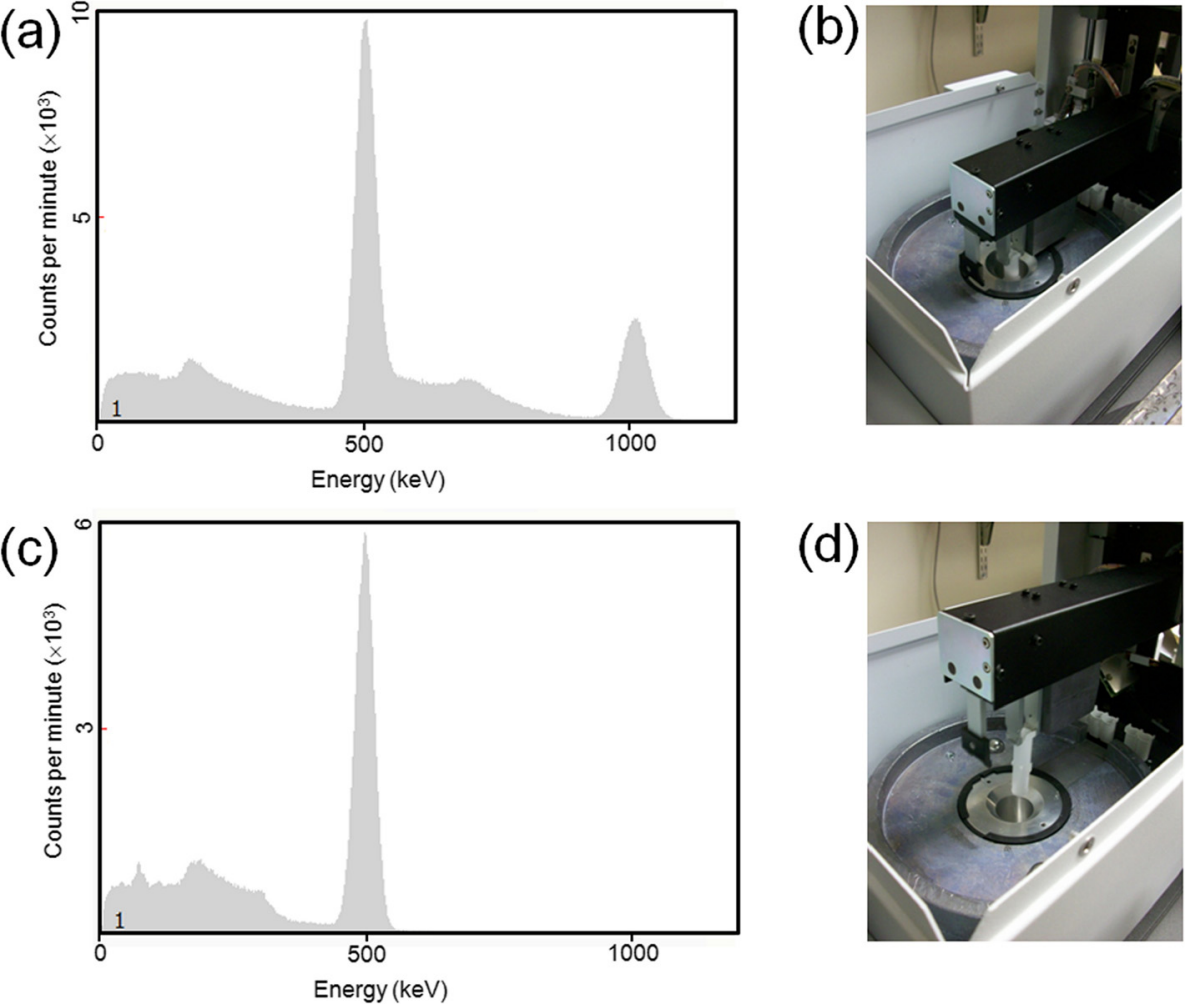
^18^F energy spectra measured with the 2480 Wizard^2^ gamma counter. When the source was located in the conventional position, inside the well **(b)**, a peak at 511 keV and a coincidence sum peak at 1,022 keV were seen **(a)**. When the source was positioned (for illustrative purposes) just outside the well **(d)**, it was not possible for corresponding annihilation photons to be simultaneously measured and, as a result, the 1,022-keV coincidence sum peak was absent from the spectrum **(c)**. Note that the photographs were taken with the shielding removed to show the two different positions of the (white) source holder.

The instrument under evaluation was a commercial well-type gamma counter (2480 Wizard^2^, PerkinElmer, Waltham, MA, USA) that consisted of a single 75-mm-diameter, 80-mm-high NaI(Tl) crystal with a 33-mm-diameter, 60-mm-deep hole. The detector was surrounded by 50 to 75 mm of lead shielding. Radioactive samples were individually transported from test tube racks and positioned inside the well of the detector under automatic control. Unless otherwise stated, the radioactive samples were in 0.3 mL aqueous solution, contained within 10-mm (inner) diameter glass test tubes. Data acquisition proceeded according to previously defined counting protocols that were identified using a bar code read from the side of the rack. At 511 keV, the energy resolution of the system was approximately 8.6% full width at half maximum. Unless otherwise stated, positron-emitting isotopes were counted using a ±20% energy window (409 to 613 keV) that was centered on the 511-keV peak. This energy window did not include the coincidence sum peak (see Figure 1), although scatter from this higher peak was not entirely excluded.

Count data were acquired for 1 min per sample unless otherwise stated in the text. The results of each counting procedure were standardized by compensating for the acquisition duration and expressed as CPM. These data were decay-corrected with reference to the start of the first sample in a multi-sample run; detector deadtime losses were corrected using the manufacturer’s proprietary algorithm; and a correction for background radiation was applied. In addition, the CPM data were converted to units of activity (Bq) using isotope-specific efficiency factors that will be described in subsequent sections.

### Deadtime correction accuracy

Count rate performance was assessed by repeatedly counting an ^18^F source as it decayed over 8 half-lives. The radioisotope was in aqueous solution and was contained within a sealed test tube to avoid evaporation losses. A source activity of 1 MBq at the time of initial counting was measured using a radionuclide calibrator (CRC 15W, Capintec, Ramsey, NJ, USA) that had, itself, been calibrated for ^18^F using a ^68^Ge/^68^Ga reference source that was traceable to an NIST standard [10]. Seventy measurements were made over a period of 15 h. In order to assess the accuracy of the system’s correction for count losses, deadtime-corrected gamma counter data were plotted as a function of source activity. The source activity at the time of the gamma counter measurements was determined analytically based on radioactive decay of the initial radionuclide calibrator measurement. A least-squares technique was used to fit a linear function to the low count rate data (under 100,000 CPM), under the assumption that these data were minimally affected by count losses. The differences between the measured gamma counter data and an extrapolation of the linear fit were used to characterize the accuracy of the system deadtime correction.

### Background correction accuracy

The effectiveness of the system’s shielding and background correction was assessed by positioning a high-activity positron source outside the detector and recording the number of photons that penetrated the shielding. For this experiment, two separate test tubes were prepared. One test tube was empty and contained no radioactivity. The other contained ^18^F and had an initial activity of 440 kBq. The test tubes were placed in adjacent positions in a rack and were counted sequentially. In this way, the empty test tube was counted with the high-activity source positioned so as to accurately reproduce a typical counting arrangement. Multiple paired measurements (*n* = 27) were made as the ^18^F decayed in order to obtain data over a range of activities. All data were background-corrected by automatically subtracting a background estimate that was measured at the time of instrument calibration, 18 CPM for the energy window used in this case. This background estimate was not necessarily performed under the same experimental conditions as a typical counting run and the above experiment assessed the effectiveness of the shielding and the applicability of this simple correction.

### Stability

Basic system performance was assessed by repeatedly counting a ^68^Ge/^68^Ga source (50 × 1 min) and performing a chi-square test to assess the consistency of the data with a Poisson distribution. Long-term stability was assessed by counting the same ^68^Ge/^68^Ga source on multiple occasions (*n* = 68) over a period of 100 days. The laboratory temperature was maintained at approximately 20°C, and measurements were made at arbitrary times during the day. The source activity was approximately 5 kBq at the time of the first measurement. Data acquisition was performed using identical parameters and involved a 10-min counting period to reduce statistical noise.

### Sample volume effects

The effect of sample volume on the relative efficiency of the gamma counter was measured using a series of experiments, each with a different radioisotope: ^18^F, ^11^C, ^125^I, ^99m^Tc, and ^51^Cr. The primary energy windows for the ^18^F, ^11^C, ^99m^Tc, and ^51^Cr experiments represented ±20% on either side of the appropriate photopeak. For those isotopes that produced multiple emissions, additional energy windows were employed. For the positron emitters, data were simultaneously acquired in a 511-keV window (409 to 613 keV), a coincidence sum peak window (920 to 1,124 keV), and a wide window encompassing both peaks (350 to 1,200 keV). The multiple emissions of ^125^I did not allow for a simple ±20% energy window of the sort used for the other isotopes. In this case, data were acquired in a lower energy window that included X-rays around 27 keV and the 35-keV gamma (17 to 41 keV). In addition, a coincidence sum peak window (47 to 71 keV) and a wide window encompassing both peaks (16 to 74 keV) were acquired. For ^99m^Tc and ^51^Cr, the energy windows were 112 to 168 keV and 256 to 384 keV, respectively.

The experiments involved preparation of a 100-μL radioactive sample in a 10-mm (inner) diameter glass test tube. Activity at the start of the experiment was less than 18 kBq in order to minimize detector deadtime. The sample was initially counted for 150 s to establish a low-noise reference count rate. Forty additional 30-s counting measurements were then performed. Between each measurement, 100 μL of non-radioactive water was added to the test tube so as to progressively increase the sample volume, while maintaining the total activity unchanged (not withstanding radioactive decay that was corrected analytically). In this way, count rate data were obtained for sample volumes between 0.1 and 4.0 mL. Each measurement was divided by the initial 150-s reference measurement in order to estimate the relative efficiency with respect to a 100-μL sample. All experiments were repeated twice on different days using separate sample preparations.

### Efficiency

The efficiency of the gamma counter for ^18^F was measured using two methods. The standard method was based on ^18^F activity measurements obtained using a radionuclide calibrator and an alternative method involved an implicitly NIST-traceable ^68^Ge/^68^Ga source.

The ^18^F procedure involved the following steps. An ^18^F sample was prepared in a 0.5-mL volume within a 3-mL plastic syringe. The activity was measured using a radionuclide calibrator (CRC 15W, Capintec), and all subsequent measurements were decay-corrected back to this reference time. The calibration setting for ^18^F (#484) had been previously determined using an NIST-traceable ^68^Ge/^68^Ga mock syringe source that had been cross-calibrated for ^18^F (X-Cal, RadQual, Weare, NH, USA). The radioactive sample was transferred from the syringe and mixed with approximately 30 mL of water in a closed container. Residual activity in the syringe was measured and subtracted from the original measurement to determine the amount of activity in the approximately 30-mL solution. The exact volume of the radioactive solution was determined by weighing the container before and after filling using an accurate balance (XS105, Mettler Toledo, Columbus, OH, USA) and an assumption of 1 g/mL for the density of water. In this way, the activity concentration (Bq/mL) in a stock solution was accurately measured. Five 0.3-mL samples were pipetted from this solution and transferred to five glass test tubes with stoppers. The exact volumes of the radioactive samples in each test tube were determined by weighing each tube before and after filling. From the volume of each sample and the activity concentration of the stock solution, the activity in each test tube was determined (approximately 5 kBq). The samples were each counted for 1 min using the 409- to 613-keV energy window with background, deadtime, and decay corrections applied. The efficiency of the gamma counter for ^18^F was determined by dividing the gamma counter data (CPM/60) by the activities in the samples (Bq). The entire measurement procedure was repeated on three separate occasions.

An alternative approach involving an NIST-traceable ^68^Ge/^68^Ga source could potentially allow for a much simpler experimental procedure. The feasibility of this approach and the effect of the different decay characteristics of ^18^F and ^68^Ge/^68^Ga were explored as follows. The experiment involved a source consisting of a 0.0096-g active matrix of ^68^Ge/^68^Ga that had been mounted towards the base of a gamma counter compatible, 12-mm-diameter, 75-mm-long plastic rod (model BM08, RadQual, Weare, NH, USA). The source activity was traceable to an NIST standard and was known with a 95% confidence level of 3.76%. At the time of the experiments, the activity was approximately 1.3 kBq. This sample was counted using the 409- to 613-keV energy window with corrections for background, deadtime, and decay. The efficiency of the gamma counter was calculated for ^68^Ge/^68^Ga, based on the gamma counter measurements and the NIST-traceable activity of the source. As with the ^18^F experiments, the ^68^Ge/^68^Ga measurement procedure were repeated on three separate occasions.

## Results

### Deadtime correction accuracy

**Figure 2:**
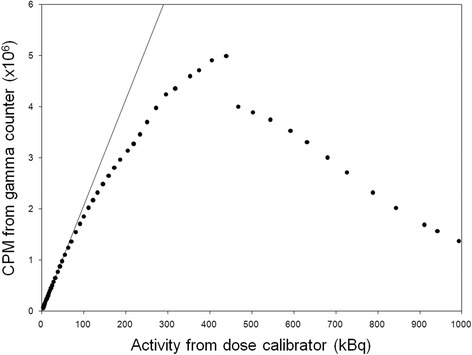
Deadtime-corrected gamma counter CPM data as a function of activity in a decaying ^18^F sample. The solid line indicates a linear fit to low count rate data and represents an idealized response under the assumption of perfect deadtime correction.

Figure 2 shows deadtime-corrected CPM data as a function of radioactivity. The solid line indicates a linear fit to 18 data points below 100,000 CPM. The difference between the measured data and the linear function was no more than 0.4%, confirming that count losses were negligible in this range. The peak count rate was 5 million CPM, although the ideal response denoted by the linear function indicates a substantial loss of accuracy at these high count rates. Five percent error was measured at 1.2 × 10^6^ CPM (63 kBq); 1% error was measured at 450 × 10^3^ CPM (22 kBq). The maximum count rate typically encountered during routine use of the instrument varies greatly between PET research protocols, but it is usually well below the rate that gives rise to 1% error.

### Background correction accuracy

**Figure 3:**
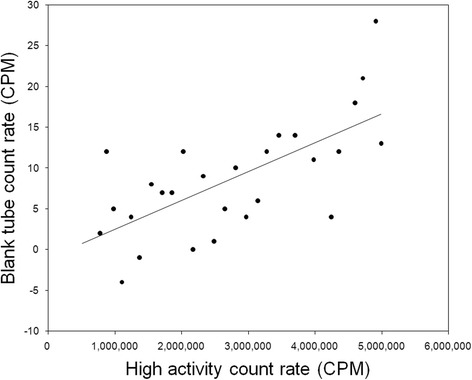
Blank tube CPM data were measured with a high-activity ^18^F source nearby. The high-activity source was also counted and corresponding data are plotted. Multiple measurements were made as the source decayed from its initial activity of 440 kBq. All data were background-corrected which accounts for the negative points among the blank tube CPM data.

Figure 3 shows blank tube CPM data plotted against the high-activity CPM data. Note the large difference in the scales of the measurements. The solid line denotes a linear fit to the data and has a slope of 3.53 × 10^−6^. These data confirm extremely low penetration of the shielding for 511-keV photons and indicate that the system’s simple background subtraction leads to a negligible loss of accuracy over a wide range of activities.

### Stability

**Figure 4:**
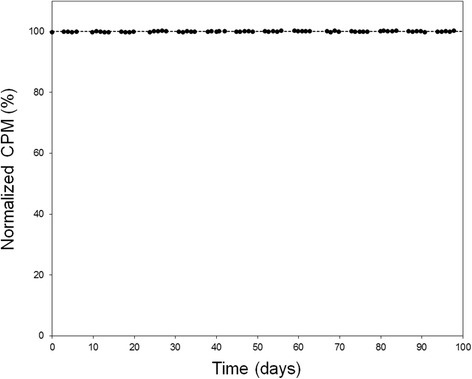
System stability assessed by repeated measurements of a ^68^Ge/^68^Ga source over a 100-day period. The data were corrected for radioactive decay with the first measurement being taken as the reference time. The dashed line indicates the mean of the data which are shown normalized to an arbitrary value of 100.

For 50 measurements and probability levels of 0.05 and 0.95, respectively, the upper and lower critical values of chi-square are 67.5 and 34.8. The measured value of chi-square was 55.7, indicating that the data are consistent with the Poisson distribution. Figure 4 shows the stability of the system over an extended period of time. Each data point was corrected for decay with reference to the time of the first measurement and normalized such that the mean of all data had a value of 100. The coefficient of variation of the measurements was 0.13%, confirming a highly stable measurement system.

### Sample volume effects

**Figure 5:**
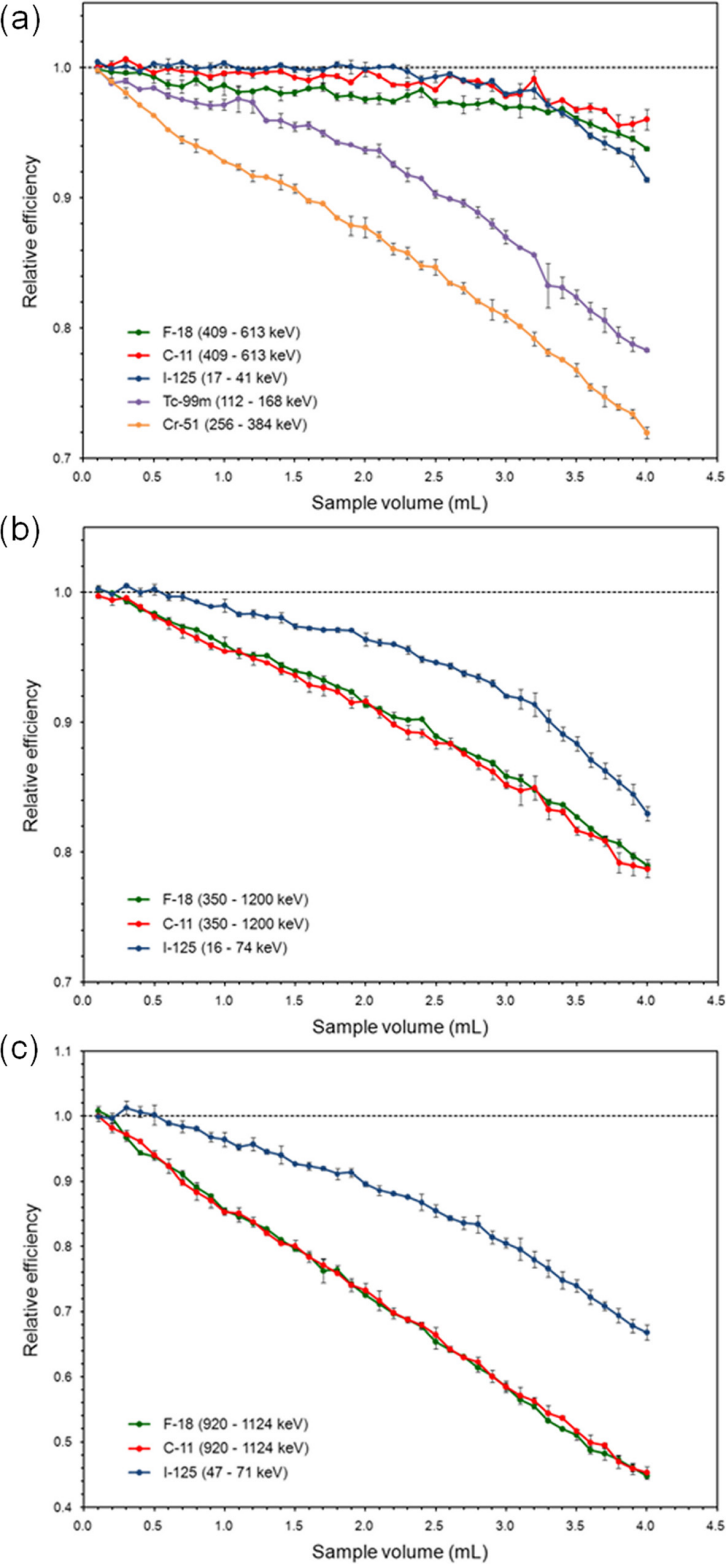
Relative efficiency as a function of sample volume for five different isotopes: ^18^F, ^11^C, ^125^I, ^99m^Tc, and ^51^Cr. Energy windows are indicated in the figure. **(a)** Data acquired in a window centered on the lower photopeak; **(b)** data acquired in a wide window encompassing both the lower photopeak and the coincidence sum peak; and **(c)** data acquired in a window including only the coincidence sum peak. The dashed lines indicate unity and are intended to aid interpretation of the data.

Figure 5a shows relative efficiency as a function of sample volume for all five isotopes when counted on their respective lower energy photopeak windows. For a sample volume of 4 mL, the relative efficiencies were 0.96, 0.94, 0.91, 0.78, and 0.72 for ^11^C, ^18^F, ^125^I, ^99m^Tc, and ^51^Cr, respectively. These 4.0-mL samples filled the test tube to a height of approximately 50 mm. Figure 5b,c shows ^18^F, ^11^C, and ^125^I data acquired in a wide window and in a window centered on the coincidence sum peak, respectively.

### Efficiency

Table 1 shows the efficiency of the gamma counter for both ^18^F and ^68^Ge/^68^Ga. Differences in the positron fractions of the two isotopes contribute to the different efficiency measurements. The last column of Table 1 indicates the efficiency per positron decay and allows for a more direct comparison of the two experimental procedures.

**Table 1:** **Experimental measurements of gamma counter efficiency (mean ± SD) for** ^**18**^ **F and** ^**68**^ **Ge/** ^**68**^ **Ga**

**Isotope**	**Efficiency (%)**	**Positron fraction**	**Efficiency (%)/positron fraction**
^18^F	34.4 ± 0.18	0.97	35.5 ± 0.18
^68^Ge/^68^Ga	30.1 ± 0.07	0.89	33.8 ± 0.07

Both isotopes were measured using a 409- to 613-keV energy window.

## Discussion

Improved standardization of methodology encourages more consistent use of PET across different centers and aids comparison or combination of data from multiple sites. Long-lived ^68^Ge/^68^Ga standards, traceable to a national metrology institute, have been introduced for radionuclide calibrators, and these sources have helped standardize ^18^F activity measurements [10]. Given that PET scanners are usually calibrated using ^18^F, improved standardization of radionuclide calibrators is expected to give rise to more accurate and more standardized PET data. In the research environment, the need for accurate cross-calibration extends to the NaI(Tl) gamma counter which is a critical component of many quantitative PET procedures. In this paper, we report our experience using traceable ^68^Ge/^68^Ga sources that are specifically optimized for the gamma counter, with the aim of improving quantitative accuracy and standardization. Although ^68^Ga and ^18^F both decay via positron emission, differences in their decay characteristics are expected to lead to different gamma counter efficiencies, and these data are shown in Table 1. After accounting for the different positron fractions of each isotope, the efficiencies of ^18^F and ^68^Ge/^68^Ga were found to be 35.5% ± 0.18% and 33.8% ± 0.07%, respectively. The different positron energies and single-photon gamma emissions associated with ^18^F and ^68^Ga could potentially contribute to the different efficiencies of the two isotopes. However, another factor is suggested by the work of Fitzgerald et al. [11] in which they recommend a 4% adjustment to the primary standardization of ^18^F. If the radionuclide calibrator were adjusted to account for this more accurate standardization, ^18^F activity measurements would increase by 4% and the efficiency of the gamma counter would change from 35.5% ± 0.18% to 34.1% ± 0.17%. The accuracy of the ^68^Ge/^68^Ga measurement is not affected by the ^18^F adjustment, and when the positron fractions are taken into consideration, the efficiencies of the two isotopes are seen to be in very close agreement: 34.1% ± 0.17% for ^18^F and 33.8% ± 0.07% for ^68^Ge/^68^Ga. This implies that long-lived ^68^Ge/^68^Ga sources that are traceable to a national metrology lab can help standardize gamma counter calibration for ^18^F and potentially for other isotopes such as ^11^C. Note that the greatly simplified experimental procedure afforded by the traceable ^68^Ge/^68^Ga source compared to the elaborate and error-prone procedure required for ^18^F is reflected by the smaller standard deviation of the ^68^Ge/^68^Ga efficiency data. Although we do not have enough experience at this stage to comment, the quoted precision of the ^68^Ge/^68^Ga source activity (95% confidence level of 3.76%) raises some concern for consistency at the time of source replacement.

The ^18^F efficiency discussed above was determined using a 0.3-mL sample which was the same as the volume typically used at our institution for assays of radioactive blood. While sample volume can usually be controlled, there are situations when radioactive samples can be of very different volumes. It is well known that increasing sample volume leads to a loss of efficiency [12], at least for single-photon emitters. We measured this effect for positron emitters and found that these isotopes have a useful characteristic that, to our knowledge, has not been previously described. For energy windows centered on the 511-keV photopeak (±20%), the relative efficiencies of ^18^F and ^11^C were much less susceptible to changes in sample volume than the single-photon emitters ^99m^Tc and ^51^Cr (Figure 5a). Of the single-photon emitters that were studied (^125^I, ^99m^Tc, and ^51^Cr), sample volume dependence was greatest for the higher energy emitters, possibly due to the greater likelihood of high-energy photons penetrating the edge of the crystal without being detected. This observation makes it all the more surprising that the 511-keV emissions from ^18^F and ^11^C were not more significantly affected by changes in sample volume.

**Figure 6:**
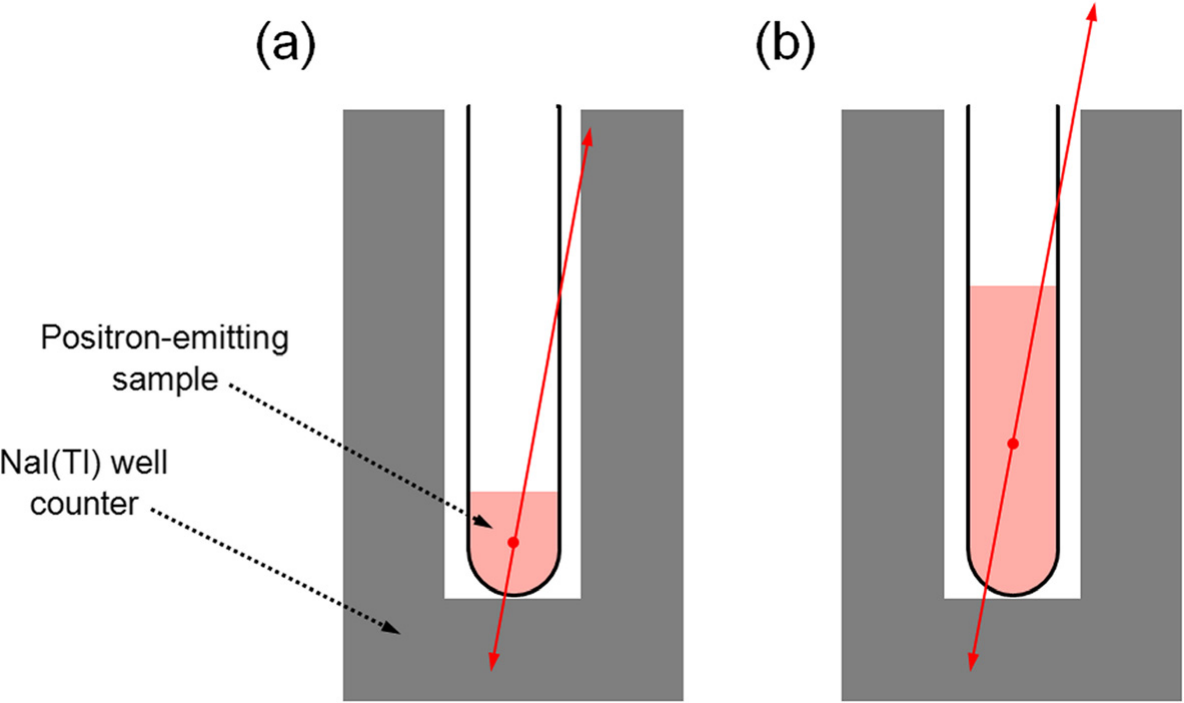
Sample volume effect. **(a)** An example pair of annihilation photons contributing to the coincidence sum peak. As the sample volume increases, there is an increasing likelihood that photons will escape from the hole at the top of the well **(b)**. Counts lost from the coincidence sum peak due to reduced geometric efficiency become single-photon events. This mechanism tends to increase counts in the 511-keV peak, partially offsetting the loss of counts due to reduced geometric efficiency.

When compared to ^125^I, the positron emitters were broadly similar (Figure 5a), although it should be pointed out that ^125^I is not usually counted in a narrow low energy window. ^125^I is a single-photon emitter that has a coincidence sum peak due to cascade emissions, and a wide window encompassing both the lower energy photopeak (consisting of 35.5-keV gamma photons and 27.5-keV X-rays) and the coincidence sum peak (around 55 keV) is typically employed [13,14]. It can be seen in Figure 5b that this wide window resulted in much greater dependence on sample volume. Indeed, Figure 5b also shows a much greater sample volume dependence for ^18^F and ^11^C when using a wide window compared to a 511-keV window (Figure 5a). Data collected in a window encompassing only the coincidence sum peak (Figure 5c) declined markedly as the sample volume increased. The loss of counts from the coincidence sum peak can be attributed to one of the two photons escaping through the hole at the top of the well. When this occurs, the remaining photon can no longer contribute to the coincidence sum peak but can potentially contribute to the lower energy peak as a single-photon event (Figure 6). In this way, counts transition from the coincidence sum peak to the lower energy peak as the sample volume increases. Counts will be lost from the lower energy window as the sample volume increases due to decreased geometric efficiency, but this loss will be partially offset by counts switching from the coincidence sum peak to the lower energy photopeak. This phenomenon may explain why the relative efficiencies of ^18^F, ^11^C, and also ^125^I are not more strongly dependent on sample volume when counted on a lower energy photopeak window.

The implication of these results is that the sample volume effect can be greatly minimized for positron-emitting isotopes by using a narrow energy window centered on the lower photopeak. The effect of coincidence, rather than co-linearity, appears to be the key, and similar results were observed for ^125^I which does not emit annihilation radiation. While greater sensitivity can be achieved with a wide window encompassing both the 511-keV peak and the coincidence sum peak, a narrow lower energy photopeak window allows greater flexibility in the sample volumes that can be reliably counted. Pre-clinical biodistribution studies involving solid tissue samples that may be of very different sizes are expected to benefit most significantly. These data are also relevant for general PET applications as they indicate that experimentally determined efficiency factors can be applicable to samples of various volumes with minimal loss of accuracy.

## Conclusions

In this paper, we assessed the technical performance of a NaI(Tl) gamma counter for use with positron-emitting radionuclides. System stability, count rate performance, detector shielding, and sample volume effects were investigated. Unexpected sample volume characteristics were noted for positron-emitting radionuclides, with very little loss of efficiency over a wide range of volumes. In addition, we showed that long-lived ^68^Ge/^68^Ga reference sources, implicitly traceable to a national metrology institute, can aid gamma counter calibration for ^18^F, potentially leading to improved accuracy and greater standardization across sites.

## Abbreviations

CPM: counts per minute
NaI(Tl): sodium iodide with thallium impurity
NIST: National Institute of Standards and Technology
PET: positron emission tomography
